# Identification of Grain Oriented SiFe Steels Based on Imaging the Instantaneous Dynamics of Magnetic Barkhausen Noise Using Short-Time Fourier Transform and Deep Convolutional Neural Network

**DOI:** 10.3390/ma15010118

**Published:** 2021-12-24

**Authors:** Michal Maciusowicz, Grzegorz Psuj, Paweł Kochmański

**Affiliations:** 1 Center for Electromagnetic Fields Engineering and High-Frequency Techniques, Faculty of Electrical Engineering, West Pomeranian University of Technology, ul. Sikorskiego 37, 70-313 Szczecin, Poland; gpsuj@zut.edu.pl; 2 Department of Materials Technologies, Faculty of Mechanical Engineering and Mechatronics, West Pomeranian University of Technology Szczecin, Al. Piastów 19, 70-310 Szczecin, Poland; pawel.kochmanski@zut.edu.pl

**Keywords:** magnetic Barkhausen noise, anisotropy, convolutional deep neural network, Short-Time Fourier Transform

## Abstract

This paper presents a new approach to the extraction and analysis of information contained in magnetic Barkhausen noise (MBN) for evaluation of grain oriented (GO) electrical steels. The proposed methodology for MBN analysis is based on the combination of the Short-Time Fourier Transform for the observation of the instantaneous dynamics of the phenomenon and deep convolutional neural networks (DCNN) for the extraction of hidden information and building the knowledge. The use of DCNN makes it possible to find even complex and convoluted rules of the Barkhausen phenomenon course, difficult to determine based solely on the selected features of MBN signals. During the tests, several samples made of conventional and high permeability GO steels were tested at different angles between the rolling and transverse directions. The influences of the angular resolution and the proposed additional prediction update algorithm on the DCNN accuracy were investigated, obtaining the highest gain for the angle of 3.6°, for which the overall accuracy exceeded 80%. The obtained results indicate that the proposed new solution combining time–frequency analysis and DCNN for the quantification of information from MBN having stochastic nature may be a very effective tool in the characterization of the magnetic materials.

## 1. Introduction

SiFe electrical steel sheets are used in many technical solutions, including electric motors or transformers [1,2,3,4,5]. There are two types of electrical sheets: non-grain oriented (NGO) and grain oriented (GO). The magnetic properties of NGO sheets do not depend on the direction [6], thus they are used in applications where uniform magnetic flux distribution is required, e.g., in electric motors [7]. The GO steels exhibit magnetic anisotropic properties. Therefore, this feature is mainly used in transformers cores. The GO sheets can be further divided into two types: conventional (CGO) and having super-oriented grain structure (High Grain Oriented—HGO) [8,9]. The HGO sheets are also referred to as high magnetic permeability steels (HiB) [10]. The production process of GO sheets is complex and includes stages such as [8,10]: secondary recrystallization, heat treatment, hot rolling, cold rolling, annealing, coating the sheets with oxide layers. As a result of this process, a texture having a majority of grains oriented towards the (110) [001] direction (referred to as the Goss texture) is expected to be achieved, leading to low power loss and high permeability in the rolling direction. On the other hand, the large grains of the texture affect forming of large magnetic domains. That results in a higher demand for energy during domain walls’ movement taking place during magnetization. To obtain better magnetic parameters, the GO sheets are often subjected to the domain refinement (DR) processing methods, such as: laser scratching (LS), plasma jet irradiation, spark ablation, grove marking, chemical treatment, or coating stress [8,10]. Exemplary, through laser treatment, losses are reduced by reducing the width of the domains. This reduces the amount of energy necessary to reorganize the domain structure in the subsequent cycles of material magnetization [10]. CGO sheets are characterized by a greater dispersion of grain orientation in relation to the rolling direction (so called tilt angle), reaching even 6–7°. In the case of HGO sheets, this angle is 2–3°. Due to such different grain structure, the anisotropic properties of those steels differ from each other. Therefore, in industrial applications, correct, non-invasive and relatively quick identification of GO steel properties becomes an important problem, requiring the introduction of methods that will quickly allow for controlled quality and verification of the properties of the tested electrical sheets.

In measurement practice, several different methods are used to study the magnetic anisotropic properties, including rotational method, torque measurement, magnetic hysteresis loop, and an induced magnetic field measurement [11,12,13,14,15]. The magnetic Barkhausen noise (MBN) has also become an perspective testing method [16,17,18,19,20,21,22,23]. The MBN phenomenon is related to the stepwise nature of the domain structure reorganization process taking place during the magnetization of the material [24]. The MBN signal is stochastic, non-stationary, and almost unique in each magnetization cycle [25]. It is frequently used to study the damage and applied stress [26,27,28,29,30,31,32,33], residual stress [22,34], or microstructure [35]. The anisotropic properties of electrical sheets were investigated with its application as well. In [19], the MBN signals measured for the range of directions of the magnetizing field, from the TD (transverse direction) to RD (rolling direction) axis, were analyzed. The highest MBN activity was obtained for the RD consistent with the axis of easy magnetization. In another paper [16], the authors investigated the influence of aspects such as texture, microstructure, and residual stress, confirming their importance for the anisotropic properties of steels. Also, the authors of [23] noted that MBN can serve as a method for analysing the magnetic anisotropic properties and texture of ferritic cold-rolled sheets. In the mentioned works, the influence of various factors on the change of the angular characteristics of classical parameters, such as energy, rms, or number of impulses of the Barkhausen noise signal, was demonstrated. A change in MBN activity in various phases of the magnetisation process was also reported. It was shown that the sequential examination of the MBN signal allows for the analysis of the reorganization of the domain structure by linking the areas of activity occurring in the MBN signal [20,21,36,37]. In the works [20,21,36], a detailed analysis in time domain of the course of the MBN phenomenon was performed. The three major activity regions referring to processes of nucleation of reverse domains, 180° and 90° DWs motion, depended on the given properties of materials, were then indicated. In [37], to better observe the dynamics of changes in activity, a method of observation and parameterization of the MBN based on building the time–frequency characteristic (*TF*) using the Short-Time Fourier Transform (STFT) has been proposed by the authors. The procedure of unambiguous division of the time–frequency space into sub-periods of MBN activity has been presented. The proposed *TF*-based method made it possible to visualize not only the changes in dynamics or to determine the level of the background of the Barkhausen phenomenon during the entire magnetization period [37], but also to identify any periodic disturbances constituting a constant contribution to the measured signal and disturbing the obtaining of information about the investigated anisotropy from the MBN signal [38].

Even though the use of the proposed *TF* analysis is a good expansion of classical methods and allows for a broad simultaneous analysis of many aspects [37,38], the correct interpretation of the MBN signal is still complex and often requires the analysis of many features describing the observed characteristics. Thus, in the context of developing automatic identification procedures for the state of the tested material few aspects remain. The problem of compiling the features vectors describing the studied signals, and then selecting their adequate representation, especially in the context of the analysis of the instantaneous dynamics of the MBN phenomenon, must be considered. The database of features ultimately has a decisive influence on the effectiveness of the entire method. The second problem is the selection of appropriate time ranges for MBN activity analysis under given anisotropic properties of material. The possible solution is to use an Artificial Neural Networks (ANN). They have been proven to be successful in various applications, i.e., in the use to Structural Health Monitoring (SHM) [39] or to damage detection [40]; however, they do not allow to fully avoid the described problematics. Recently, due to the innumerous extension of capabilities of computing machines, the deep learning methodology, that were created on the basis of ANN, brings new perspective and shows great potential to identification procedures’ automatization. The deep neural networks make it possible to create a system that enables the transition from expert to algorithmic description without defining the classification conditions, which allows for the elimination of possible human-based errors and the automation of the decision-making process [41]. They are used in various industries. As well, they meet the rapidly growing interest in their application in non-destructive testing, for example, to control the condition of building structures [42,43], railway structures [44], or testing the properties of materials [45]. Deep networks based on autoencoders have also been successfully used to analyse the information contained in the time series of the Barkhausen noise signal for automatic and precise estimation of magnetocrystalline energy [46]. Due to the possibilities of Deep Neural Networks (DNN) and their sensitivity to even subtle changes in the input data, it is possible to apply them to the analysis of the MBN signal obtained from electrical steel sheets. Moreover, considering the acquisition of time–frequency characteristics in the form of two-dimensional distributions, convolutional neural networks (CNNs) stand out with a high application potential [41].

Therefore, to avoid the above described problems in this study, the new approach to MBN analysis based on STFT transformation and the use of deep convolutional neural networks (DCNN), to identify the type of GO sheet and to evaluate its magnetic directions, is proposed. The aim of the work is to investigate the possibility of building identification procedure sensitive to even subtle changes in the dynamics of the MBN signal. Thus, STFT spectrograms, allowing simultaneous consideration of MBN phenomenon dynamics expressed both in time and frequency, are proposed as input data of deep neural network model with the convolution layer. First, the structure of the DCNN was adjusted in preliminary experiments. Then, the DCNN was used to distinguish sheets with different anisotropic properties (obtained using different surface engineering methods) and to recognize the magnetic angles between the rolling and the perpendicular to the rolling directions. Additionally, the predations updating procedure, allowing improvement of the DCNN performance, was also introduced. Finally, the assessment of the proposed approach accuracy as a function of various angular resolutions applied during testing was carried out and the optimal conditions were discussed.

## 2. Measuring Samples and Characterization of Materials

Measurements were made for various types of SiFe grain-oriented steel sheet having different properties manufactured by two producers (marked as #1 and #2). The list of sheets with their parameters is presented in Table 1.

To determine the influence of the GO electric steel type and various possible techniques for modelling their magnetic properties on the dynamics of the MBN phenomenon, two series of samples were investigated. In the case of the materials of the first producer, the experiments were carried out for a series of samples made of CGO sheets of different thickness. The set also contained the HGO sheet with a thickness corresponding to the thinnest CGO one in the set. In addition, this series of samples also included both CGO and HGO sheets subjected to domain refinement process using the laser scribing technique. In the case of the second series, the tests were carried out for three samples made of CGO sheet. The first represented the manufacturer’s base material. The second sample of this series was obtained by mechanical removing of (decoating) the oxide layer covering the steel surface. To compare the possibilities of modelling the anisotropic characteristics of electrical steels, the nitriding process was additionally carried out, thus obtaining the third sample of the series. The nitriding was conducted with a gas method in a mixture of ammonia (approximately 50 vol.%) and products of its dissociation at temperature of 570 °C for 3 h.

Samples for metallographic examinations were precisely cut than mounted in a conductive resin (Polyfast, Struers, Ballerup, Denmark) and mechanically ground and polished using a 0.25-µm diamond suspension. This was followed by polishing using a fine silica suspension. Low angle (7°) argon ion milling (Flat Milling System IM-3000, Hitachi, Naka, Japan) was the final stage of the sample preparation procedure for EBSD (electron backscattered diffraction) and X-ray microanalysis.

The samples were examined using an FE-SEM (Field Emission Scanning Electron Microscopy) SU-70 microscope (Hitachi, Naka, Japan) equipped with EDS (energy dispersive spectrometry) X-ray microanalysis UltraDry X-ray detector and EBSD (Electron Backscattering Diffraction) QuasOr. The EDS and EBSD components were integrated under the NORAN™ System 7 from Thermo Fisher Scientific (Madison, WI, USA).

EBSD crystal orientation and EDS measurements were acquired at an accelerating voltage of 20 and 15 kV, respectively.

The X-ray diffraction (XRD) phase analysis was conducted using X-ray tube CuKα, operating at the voltage of 35 kV, current 45 mA and a Bragg–Brentano geometry (X’Pert–PRO, Panalytical, Almelo, The Netherlands). The applied step of the goniometer was 0.05°, and the acquisition time was 200 s. The data was processed using X’Pert HighScore (v. 2.2.1) software provided by Panalytical.

All results obtained during the conducted by authors material characterization were presented in following figures and tables of this section.

**Figure 1:**
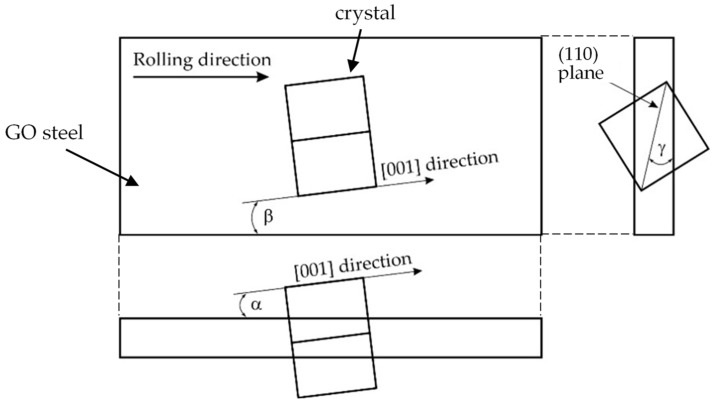
A schematic representation of a single crystal misorientation angles in relation to the ideal Goss texture presented in three orthogonal planes.

The EBSD results showed that the samples H_23#1_ and H_23LS#1_ were characterized by the smallest deviations from the ideal Goss texture (Figure 1, Table 2) in the beta angle (tilt angle).

The silicon content in all tested materials was similar and amounted to approximately 3 wt.%. (Table 3).

**Figure 2:**
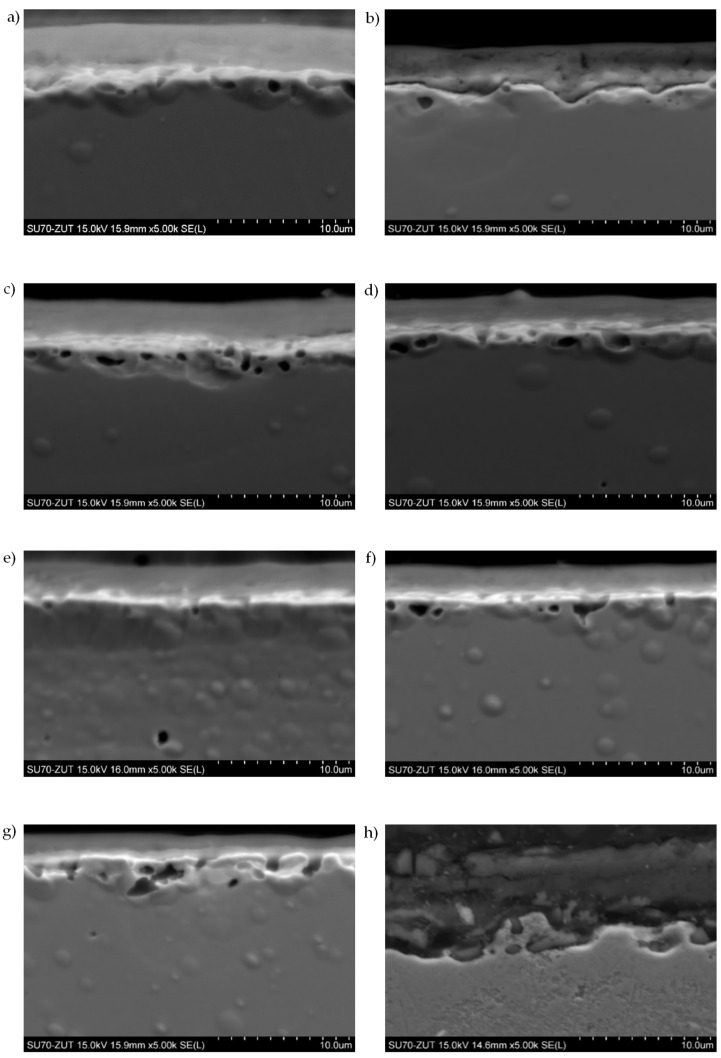
Secondary electron images of oxide layers on samples obtained during the carried-out tests for: (**a**) C_23#1_, (**b**) H_23#1_, (**c**) H_23LS#1_, (**d**) C_27#1_, (**e**) C_27LS#1_, (**f**) C_30#1_, (**g**) C_30LS#1_, (**h**) C_27#2_.

**Figure 3:**
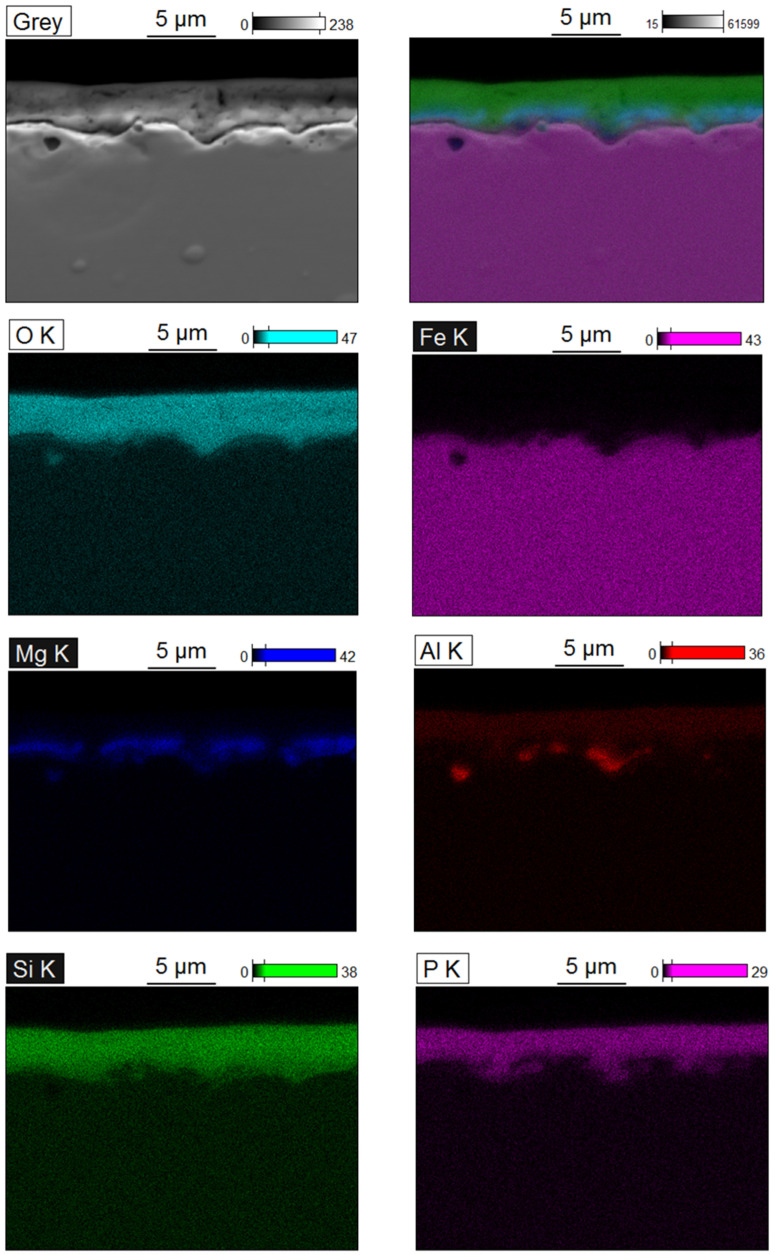
Elemental mapping of cross-section of surface layer of H_23#1_ obtained during the carried-out tests. EDS.

The measured thickness of the surface oxide layers (Figure 2 and Table 4) was varied and ranged from about 3 to 9 µm depending on the sheet manufacturer. The microstructure of the oxide layers of the examined steel sheets (see Figure 2) was similar for all samples and was typical for modern electrical sheets [47]. For all samples, the outer zone of the oxide layers was rich in aluminium, silicon and phosphorus. The inner zone contained mainly magnesium and oxygen (Figure 3).

**Figure 4:**
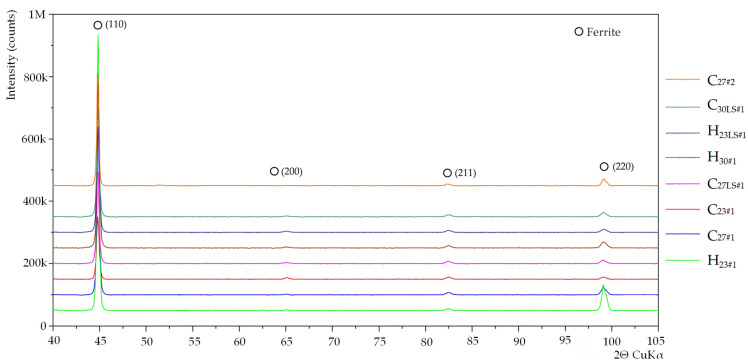
Diffraction pattern acquired from samples H_23#1_, C_27#1_, C_23#1_, C_27LS#1_, C_30#1_, H_23LS#1_, C_30LS#1_ and C_27#2_ after removing the oxide layer.

**Figure 5:**
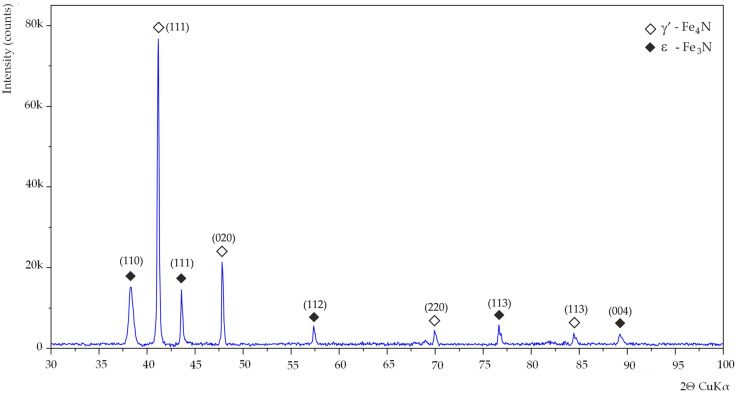
Diffraction pattern acquired from nitrided C_27N#2_.

The diffraction patterns for all tested samples were similar (Figure 4). XRD tests of the sheets carried out after the removal of the oxide layers showed a significantly increased intensity of the diffraction peak from the planes (110), which is typical for steel with a Goss texture. A significantly higher intensity of the peak assigned to planes (110) was observed for sample H_23#1_, what is consistent with the EBSD result, where the smallest misorientation angle γ was measured (Table 2). X-ray diffraction examinations of the nitrided sample (Figure 5) showed a 2-phase structure. The presence of γ’—Fe_4_N and ε—Fe_3_N nitrides has been identified. The ferrite peak was not observed, which proves the nitriding to entire thickness of the sheet.

## 3. Measuring System and Measurements Results Processing

**Figure 6:**
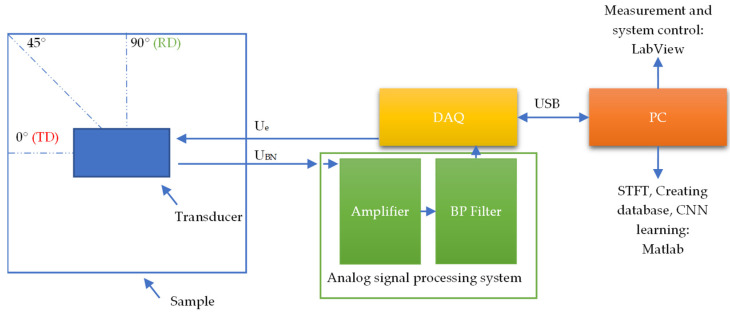
The diagram of the measuring system and procedure.

The diagram visualizing the configuration of the system along with the measuring procedure is presented in Figure 6. The measurements were made for 51 angular settings of the MBN measuring transducer. The transducer rotation angle was ranging from 0° to 90° at a resolution (angular rotation step) of 1.8°, where 0° corresponds to a situation where the transducer magnetizes the sample along the TD direction. The measurements were performed using an automated positioning system and a signal acquisition system controlled by a computer. Data acquisition was carried out by a personal computer equipped with a DAQ card (NI-PCI-6251). The sine-shaped excitation signal having a frequency of 10 Hz was utilized. The resulting current amplitude value was 0.37 mA, which corresponded to a field strength of 1.6 kA/m. The sampling frequency was equal to 250 kHz. The unit of combined low and high pass filters created a pass band ranging from 2 kHz to 100 kHz. The detailed description of the transducer, system and measurement parameters as well can be found in the previous papers [27,37,38].

The STFT was used to transform the MBN signal from the time domain into the time–frequency one in the form of spectrograms *S*_BN_*(t, f)*. In the transformation process, the Kaiser function with a size of 512 points was used, which resulted into a resolution in time of 512 µs and in frequency of 488 Hz. Details of the STFT transformation procedure for the analysis of the MBN phenomenon have been described by the authors in their previous papers [26,37,38]. Since the highest activity of MBN was observed in the lower ranges of the spectrograms, the utilized frequency band was limited. Considering that the variance for the 0–72 kHz range is approximately 30-times greater than the variance for the remaining range, the 72 kHz was chosen as the high frequency limit of the band. Hence, the applied limitation does not affect the loss of important information while allows a significant reduction in training time and the operation of the entire procedure in further stages. Finally, the spectrograms amplitude distributions |*S*_BN_(*t*, *f*)|^2^ matrix, representing the described frequency range was used as the input data to the designing neural network.

**Figure 7:**
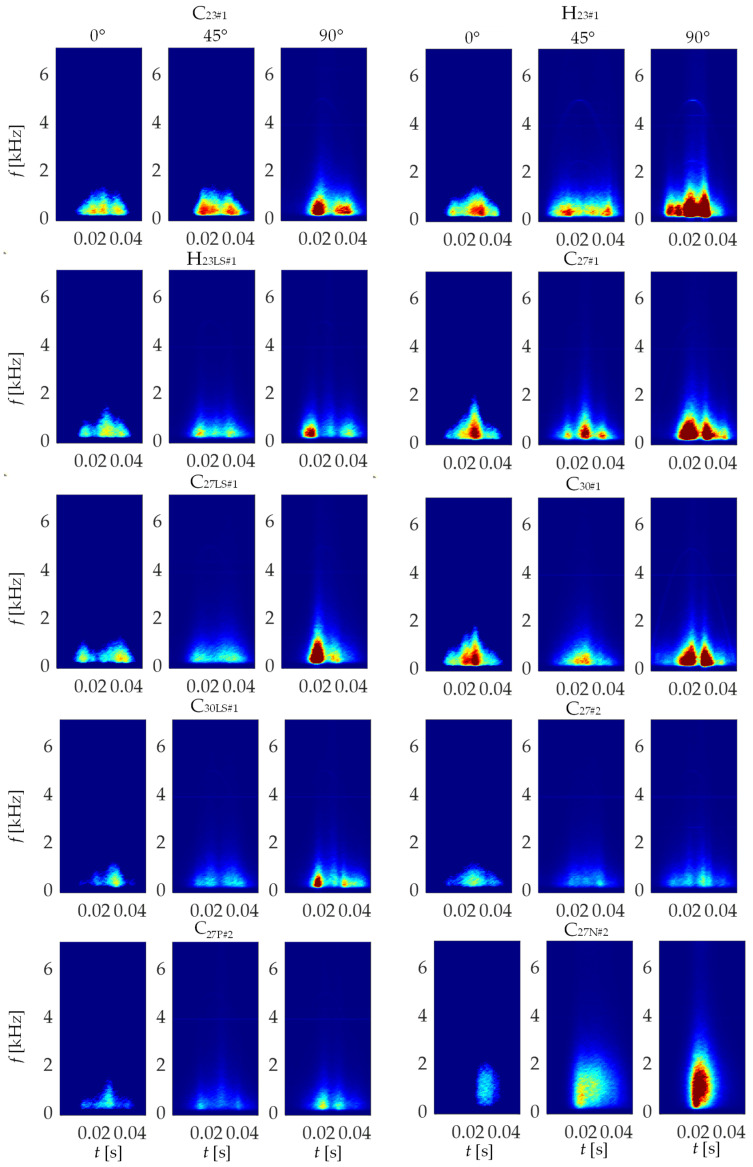
Selected amplitude of STFT spectrograms |S_BN_(*t, f*)|^2^ obtained for three different angles between magnetization direction and the TD direction.

Figure 7 presents exemplary amplitude of the STFT spectrograms |*S*_BN_(*t, f*)|^2^ obtained for all tested samples and for three magnetization directions: 0, 45, and 90 degrees. All results were shown in the common range and the colour map was expressed in logarithmic scale. As it can be seen, the highest spectral power density values over the entire magnetizing period were obtained for the HGO sheet (H_23#1_). Significant activity is visible not only for the 90° angle, as is the case of the rest of the samples, but also for the 0° and 45° angles as well. In the case of CGO sheets (C_23#1_, C_27#1_, C_30#1_) it can be noticed that for the C_23#1_ the spectral power density, within the highest MBN activity areas, achieves generally lower values ranges on the spectrograms than for the C_27#1_ and C_30#1_. It can also be seen that for the thicker sheets (C_27#1_ and C_30#1_), the MBN activity is more concentrated near the centre of the magnetizing period and encompasses higher frequency bands. It can be explained in the context of highest rate of dispersion of grain orientation of sample C_23#1_. The misorientation results in deterioration of the bulk magnetic properties of electrical steels (i.e., decrease of magnetic permeability and increase of coercivity) [48]. Thus, generally the lowest activity can be noticed for steel C_23#1_. In the case of CGO sheets subjected to laser scribing, higher values of power density and a larger area of the highest activity are observed for the thinner sheet (C_27LS#1_) than for the thicker one (C_30LS#1_). In the case of the C_27#2_ sheet, the removing of the insulating oxide layer (decoating) resulted in general increase of spectral power level in successive activity areas concentrated around *t* = 0.02 s and *t* = 0.03 s. The decoating affects the increase in average 180° domain width being a result of stress release, which was introduced to the steel by a coating process. In consequence, the higher rate of change in magnetization is obtained, affecting the increase in MBN activity [49]. However, there is a clear change in the *TF* characteristics for the sample obtained in the nitriding process (C_27N#2_). For the 0° angle, the activity in the *TF* space is mostly concentrated from about 0.025 to 0.035 s. Then, for the 45° angle, there is a visible shift and an increase in activity from about 0.02 to 0.04 s. For the 90° angle, a compact area (from about 0.02 to 0.03 s) with very high spectral power density values was obtained. Considering steel having the same thickness (C_23#1_, H_23#1_ and H_23LS#1_), it can also be noticed that for laser scribed HGO (H_23LS#1_) sheet the three areas of activity can be distinguished [37,38], while compared to the CGO (C_23LS_) and the HGO (H_23#1_) the lowest values of the power density regardless of the magnetization angle was obtained for it as well. The scribing process introduces local stress and further results in reduction of 180° domains width and population of 90° ones as well. This lead to lower rate of change in magnetisation and thus lower MBN activity [48,50].

Based on the given distributions of |*S*_BN_(*t*, *f*)|^2^, one can clearly see differences in the course of the instantaneous dynamics and the level of activity of the MBN depending on the steel grade and machining technique. Observation of the course of MBN with the use of time–frequency spectrograms enables the visualization of various stages of the changes taking place in a broader sense. A detailed interpretation of the changes in the domain structure taking place during the process of magnetization requires further sequential analysis. However, in the approach proposed in this paper, due to the properties of the deep neural network, the adequate definition of the human-chose features characterizing the spectrograms and reflecting different properties of the examined material is not the aim of the work, as the features are being autonomously coded in training process of the neural network.

## 4. Deep CNN-Based GO Steel Identification Procedure

In this section, an approach for identification of SiFe steel type and the measuring magnetic conditions using the deep convolutional neural network is described. Information on database division, an algorithm improving the efficiency of the neural network and parameters determining the accuracy of the prediction model are also given.

### 4.1. Convolution Neural Networks

Convolutional neural networks have emerged from study of the human visual cortex structure. The neurons making up this structure define local reception fields. Individual neurons respond to different stimuli (elements). Behind the neurons that recognize simpler shapes are others that recognize more complex image elements. Similarly, CNNs also consist of many layers, including interconnected layers or activation functions, but the most important layer is the convolution one [51]. Due to their properties, CNN networks find many applications in computer vision and image recognition [41]. Applications in object recognition and tracking [41], medicine—for imaging diagnosis of diseases [52] or in technology—for the assessment of the state of structures [42,45,53,54,55,56] or design [57], and testing the properties [53] of materials, comprise a special area. For example, in [52], the effectiveness of detecting carious lesions was on average between 82% and 89%. In technical applications, equally high accuracy has been achieved in the use of the CNN network in the process of detecting material anomalies in the nano scale [53] or the production of microstructural materials [57]. The CNN are also used in the field of non-destructive testing (NDT). In [54] and [55], the CNNs have been used for the automatic defect identification, respectively, in steel elements based on magnetic imaging and in 3D printouts by thermal examination, achieving an overall accuracy of over 90%. The CNN are also used to detect changes in dynamic systems and classify their states based on STFT spectral characteristics [58,59,60]. The combination of the properties of the STFT transformation, which makes it possible to visualize the dynamics distribution of the observed system in time, together with the ability of the CNN network to sense even complex dependencies, creates the capability of distinguishing a wide range of states. For example, in the paper [58], processed seismographic images obtained as a result of STFT transformation were used. On their basis, the CNN network was trained, which classifies the assumed groups of seismological phenomena. Very good detection results were achieved for these phenomena, reaching a level of over 95%. These few examples show that the CNN can be used in many areas, and most importantly, in each of them they are significantly accurate. Therefore, in this article, they were used to distinguish the dynamics of the Barkhausen phenomenon based on the STFT distributions obtained during the experiments. Such approach, to the best authors knowledge, has not been the subject of any previous work.

### 4.2. Construction of Database and Network Structure

First, the measurement data obtained for all samples were used to build the database. Each sample was measured 10 times for each magnetizing angle position. There are 10 bursts of MBN in single measurement set for a single angle, so each unique case has the same representation in the base. The measurement data was then processed using the STFT transformation, and then the computed distributions of |*S*_BN_(*t, f*)|^2^ were divided into three subsets for the training process in the following proportions: 60%—a training set, 20%—a validation set, 20%—a testing set.

In the next stage, firstly a series of preliminary experiments were carried out, during which the impact of the neural network structure configuration and the training process parameters on the obtained accuracy was analyzed to determine the conditions allowing to achieve its highest level. This process was carried out with the use of the database described above and it concerned running the computations for over 9 × 10^3^ cases. As part of the selection of the network structure, the parameters of two basic layers, i.e., the convolutional and averaging ones, were considered. In the first case, the analysis was performed for a series of its parameters: the size ranging from 1 to 60, the stride ranging from 1 to 5, and the number of filters ranging from 1 to 128. Similarly, in the case of average pooling layer, the size and the stride parameters were ranging, respectively, from 1 to 8 and from 1 to 5. After selecting the configuration of the structure containing the single convolution layer (the resulting sequence of all layers is consistent with the description of rows 1–5 and further 10–12 of Table 5.), the combinations with two such layers were also tested (the sequence of layers as given in Table 5 lines 1–12). In the case of the second convolution layer, the size from 1 to 40, the stride from 1 to 5 and the number of filters from 1 to 128 were considered. Simultaneously, the parameters of the size and the stride of the second averaging layer took values from 1 to 2. In principle, the structure of the network was to remain relatively simple, consisting of a few layers to prevent the phenomenon of vanishing and exploding gradient [51] (in consequences of implementing too many layers) while still creating the possibility of achieving high efficiency. Therefore, further increase in the number of the convolutional layers was not considered in this work.

During the experiments the impact of the DCNN training parameters such as: the trailing algorithm type (three gradient algorithms were considered: *Adam*, *SGDM*, *RMSProp* [61]), the initial learning rate (ranging from 0.001 to 1), the mini-batch size (ranging from 1 to 32) and the number of epoch (ranging from 25 to 125) were analyzed. The final values obtained in the preliminary process guaranteeing the highest accuracy of correct identification of individual cases, are summarized in Table 5 and Table 6. The average time of the prediction process carried out for a single set of input data containing 10 records, using the standard office-class computer with 3.6 GHz AMD Ryzen 7 unit and 32 GB of RAM, does not exceed 25 ms.

### 4.3. Analysis of the Constructed Network Operation

**Figure 8:**
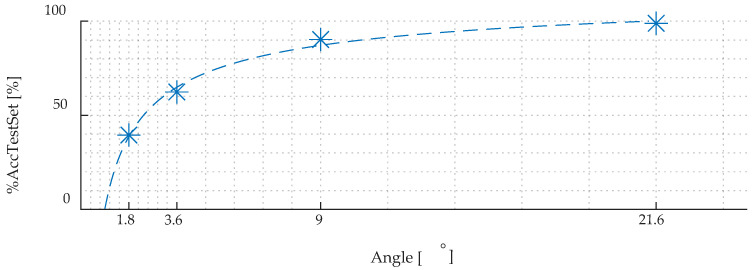
Values of the accuracy *%AccTestSet* depicted with blue star mark with approximation function (blue dash line) versus rotation step angle size.

To verify the quality of the final selected configuration of the DCNN structure and the training parameters, the feasibility of the network was examined. In these works, the influence of the different value of angular step size of the transducer on the DCNN accuracy were analyzed. For this purpose, the transducer’s rotation step between successive measurements was taking the values of 1.8°, 3.6°, 9°, and 21.6°. The angular step size affects the number of unique cases in the training set and the resolution of differentiating unique cases by the DCNN as well. By this approach, it was possible to assess the accuracy *%AccTestSet* of correct identification for different angular resolution of measurements, defined as:

(1) $$\%AccTestSet=\frac{PR}{NoR}\times 100$$

where: *PR* is the number of properly predicted records and *NoR* is the number of all records. The obtained result has been depicted in Figure 8 with the blue star mark. As it can be seen, based on the plotted approximation curve, the parameter *%AccTestSet* value increases with the increasing angular step size; however, simultaneously, the rate of the *%AccTestSet* growth decreases. The use of larger angular step allows to increase the percentage of correctly recognized types of steel and their magnetic directions (the angles of the transducer alignment with respect to TD and RD). It is due to the obvious simplification of the classification task and the reduction of possible classes (in the case of 1.8° step size there were 510 classes, while for 21.6° there were only 50). However, it is important to obtain not only highest possible classification accuracy, but also the highest number of identified direction defining, in this case, the resolution of the classification procedure. It should be assumed that the major axes of the steel are not know prior to measurement and that the transducer may be applied in any direction with respect to these axes. Therefore, it is important that the resolution of the method is as high as possible, so that the error will be as low as possible.

Performance of the constructed predictive model has been measured by using confusion matrix, which represents the rate of *true positive* (*TP*), *false positive* (*FP*), *false negative* (*FN*) predictions given by the classification model. For each class the standard parameters, *Precision*, *Recall* and *F1-Score*, where calculated from the matrix and used to present the model quality in a quantitative way [62]. The utilized quality expressing coefficients have been presented in Table 7.

The *Precision* allows one to determine the probability that the records, which are classified as positive are true positive, and thus how much one can trust the trained model by obtaining the positive prediction. The *Recall* allows to determine what percentage of positive records were qualified as correctly predicted. The *F1-Score* is the harmonic mean of two above parameters. The harmonic mean is influenced by smaller values, therefore if the parameters *Precision* and *Recall* values are close to each other, the *F1-Score* is obtaining high value as well. In the case of multiclass models, to process the assessment it is necessary to calculate the combined, so-called macro coefficients: *MacroAvreagePrecision*, *MacroAvreageRecall*, *MacroF1-Score*. Their definitions are presented in Table 7 and obtained values are presented in Table 8.

**Figure 9:**
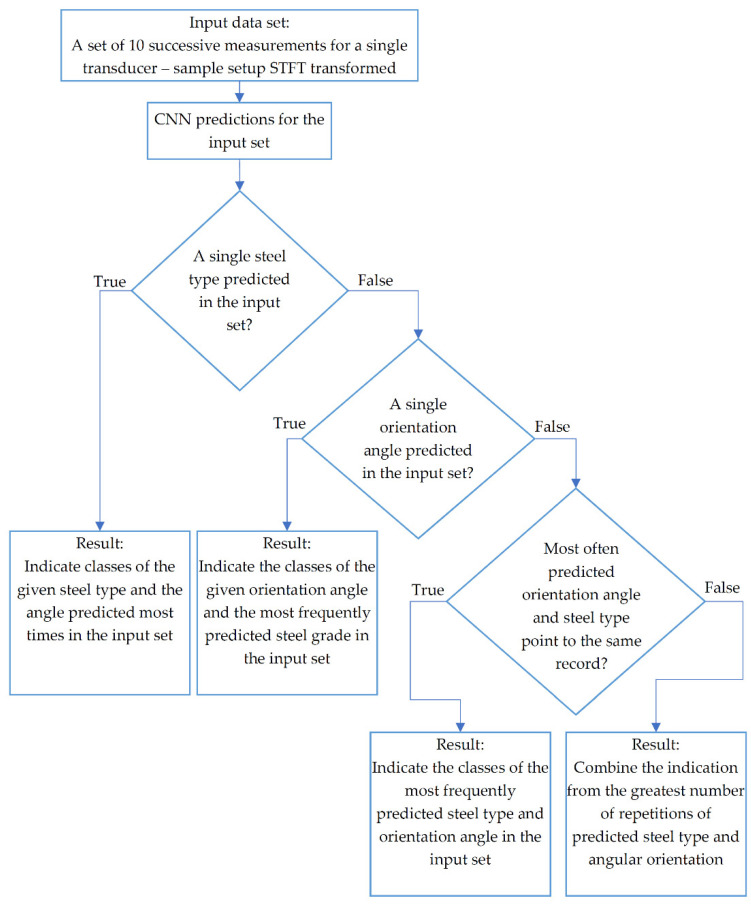
Diagram of the prediction updating algorithm.

The presented results confirm a high accuracy, exceeding 90%, for two cases with the lowest angular resolution i.e., 9° and 21.6°. At the same time, reducing the step value below 9°, results in significant drop in the accuracy, which value strongly differs from 90% in these cases. Since the measurement sequence at a given unique case is performed ten successive times and for each single measurement ten MBN burst are registered, an additional prediction updating algorithm was introduced. It allows to increase the accuracy of the correct prediction of the appropriate class by means of the probability distribution for ten measurements preformed under the same condition (for single steel sheet and single transducer orientation). The block diagram of this procedure is presented Figure 9.

The proposed procedure is based on the determination of the distribution of the number of responses in the designed network indicating individual classes of the sample type and the measurement angle. In the first stage of the algorithm, the types of electrical steel and angles have been recognized in the given set of input spectrograms are checked. Then the number of individual repetitions of a given steel types and angles in the recognized set is counted. Then, the first stage of prediction update process follows. If only one type of steel is indicated by the DCNN, the final decision is made with an indication of the angle repeated most times in the test set. When the different types of steel type are identified, the number of indications of individual angles are verified. If only one angle has been recognized, the final decision of class affiliation refers to the greatest number of repetitions of the predictions of a given type of steel. In the case when several angles have been recognized, it is checked whether the greatest number of repetitions of the angle and type of electrical steel refer to the same unique case (the same angle and the same type of steel) that is in the input data. If this is true, this record class is selected as an update. In the case when the greatest number of repetitions of a given angle and steel type indicates different unique cases, a combination consisting of those two values is created.

**Figure 10:**
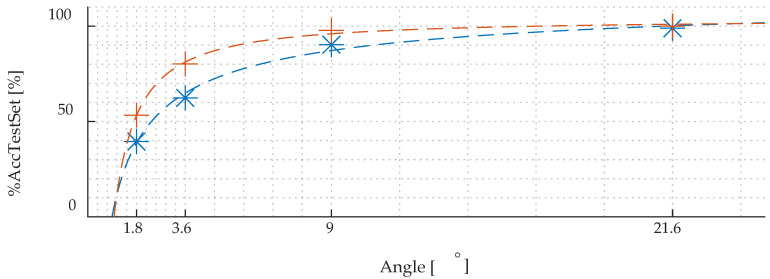
Values of the accuracy *%AccTestSet* distribution over successive angular step sizes prior (blue colour plot) and after (orange plot) application of the predictions updating algorithm.

After applying the described procedure, the assessment of the accuracy was repeated using the *%AccTestSet* given in (1). As it can be seen from the graph presented in Figure 10, the *%AccTestSet* parameter value using the update algorithm (depicted with orange colour) allows for significant increases in the accuracy of identifying individual cases by the DCNN, especially in the case of the smallest angular steps.

**Figure 11:**
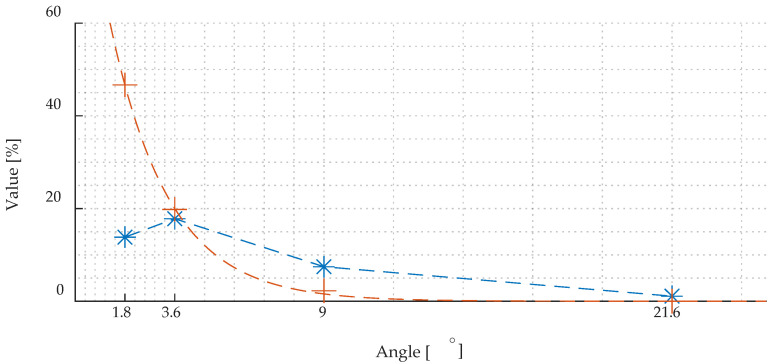
Percentage growth of parameter *%AccTestSet* (depicted with blue line) along with the deviation of it from 100% level (depicted with orange line) after applying the predictions updating algorithm.

The Figure 11 shows the growth of the accuracy of correct identification after applying the predictions updating algorithm. As can be concluded from Figure 10 and Figure 11, the use of an angular step of 3.6° allows also to obtain the highest rate of accuracy growth associated with the use of the update algorithm (exceeding the level of 80%). Additionally, the use of a resolution step equal to 3.6° is convergent to the lowest dispersion value obtained for HGO steel. Hence, for general use, the step angle equal 3.6° to predict GO steel samples and their magnetic directions is chosen as the optimal resolution.

## 5. Conclusions

The aim of this study was to investigate the feasibility of the proposed quantification procedure for information contained in MBN for distinguishing anisotropic magnetic properties of GO steels. A new approach to analysis of the MBN based on time–frequency spectrograms and the use of deep neural networks for knowledge extraction and material properties identification was proposed. The tests were carried out for a range of different steel grades. The examined group included combinations of samples having various thickness and resulted from different applied methods of production and surface engineering processing. The materials characterization was processed to obtain the basic material structure properties. The transformation of signals to the time–frequency space allowed for the visualization of the instantaneous MBN dynamics simultaneously in two component domains, creating a consistent description of the observed phenomenon in a combined 2D form. Further, the utilization of the deep convolutional neural networks allowed for autonomous searching for complex relations and building the knowledge necessary to carry out the process of distinguishing different properties of the tested materials. The presented results indicated a great potential for the development of MBN analysis methods based on automatic detection of features with the use of deep learning. The conducted work showed the possibility of detecting even subtle changes in a stochastic signal, such as the MBN, which may contribute to better understanding of several different components influencing a given course of the Barkhausen phenomenon in the examined object. Thus, after extending the scope of materials and considering a wider combination of domain refinement methods, it is also possible to obtain a tool of high generalization ability and versatility. At the same time, considering the relationship between magnetic and microstructural properties, it is expected that this method can be used not only to assess the properties of GO steels but also to a number of other applications for magnetic materials characterization as well.

## Figures and Tables

**Table 1:** The steel samples set with their parameters.

Sample Name	Sample Type	Thickness [mm]	Min. Lossiness (1.7 T/50 Hz) [W/kg]	Min. Induction [T]
C_23#1_	CGO	0.23	1.10	1.80
H_23#1_	HGO	0.23	0.95	1.88
H_23LS#1_	HGO with LS	0.23	0.95	1.87
C_27#1_	CGO	0.27	1.14	1.80
C_27LS#1_	CGO with LS	0.27	1.05	1.80
C_30#1_	CGO	0.30	1.17	1.80
C_30LS#1_	CGO with LS	0.30	1.07	1.80
C_27#2_	CGO	0.27	1.20	1.80
C_27P#2_	decoated CGO	0.27	N/A	N/A
C_27N#2_	nitrided CGO	0.27	N/A	N/A

Note: #1 and #2 in indexes refers to the producer no. 1 and no. 2 of the steel.

**Table 2:** Values of misorientation angles in relation to ideal Goss orientation. EBSD.

Sample	α [°]	Std. dev. [°]	β [°]	Std. dev. [°]	γ [°]	Std. dev. [°]
C_23#1_	26.9	0.9	4.3	0.5	4.8	0.2
H_23#1_	5.3	0.6	2.8	0.7	0.7	0.5
H_23LS#1_	4.7	1.5	2.1	1.6	9.9	0.5
C_27#1_	8.6	0.7	5.8	0.8	3.3	0.6
C_27LS#1_	1.0	0.8	3.4	1.8	4.7	0.9
C_30#1_	1.0	0.8	3.1	1.2	2.4	0.8
C_30LS#1_	3.8	0.8	3.1	2.0	3.9	1.0

**Table 3:** Silicon content in samples [wt. %]. Standard uncertainty value 0.1 %. EDS.

Sample	C_23#1_	H_23#1_	H_23LS#1_	C_27#1_	C_27LS#1_	C_30#1_	C_30LS#1_	C_27#2_
Si content	2.9	3.1	3.1	2.9	2.8	2.9	2.9	3.0

**Table 4:** Thickness of oxides layer [µm]. Standard uncertainty value 0.5 µm.

Sample	C_23#1_	H_23#1_	H_23LS#1_	C_27#1_	C_27LS#1_	C_30#1_	C_30LS#1_	C_27#2_
Thickness	4.8	3.8	4.2	4.0	2.9	3.4	3.6	9.0

**Table 5:** Final DCNN structure and configuration of successive layers.

Lp.	Layers	Size	Stride	FilterNum
1	Image Input	145 × 94 × 1	N/A	N/A
2	Convolutuon	60 × 60	1 × 1	32
3	Batch Normalization	N/A	N/A	N/A
4	ReLU	N/A	N/A	N/A
5	AveragePooling	8 × 8	5 × 5	N/A
6	Convolutuon	4 × 4	3 × 3	128
7	Batch Normalization	N/A	N/A	N/A
8	ReLU	N/A	N/A	N/A
9	AveragePooling	1 × 1	2 × 2	N/A
10	Fully Connected	N/A	N/A	N/A
11	Softmax	N/A	N/A	N/A
12	Classification Output	N/A	N/A	N/A

**Table 6:** Selected training process parameters.

Parameter	Value/Name
Training algorythm	*RMSProp*
Initial learning rate	0.005
Mini Batch Size	32
Number of epoch	75

**Table 7:** The DCNN quality assessing coefficient.

**Name of Parameter**	**Formula**	
*Precision*	$Precision=\frac{TP}{TP+FP}$	(2)
*Recall*	$Recall=\frac{TP}{TP+FN}$	(3)
*F1-Score*	$F1-Score=2\times \left(\frac{Precision\times Recall}{Precision+Recall}\right)$	(4)
*MacroAvreagePrecision*	$MacroAveragePrecision=\frac{\sum_{i=1}^{n}Precision}{n}$	(5)
*MacroAvreageRecall*	$MacroAverageRecall=\frac{\sum_{i=1}^{n}Recall}{n}$	(6)
*MacroF1-Score*	$F1-Score=2\times \left(\frac{MacroAveragePrecision\times MacroAverageRecall}{MacroAveragePrecision+MacroAverageRecall}\right)$	(7)

**Table 8:** Values of parameters measured the quality of the predictive model.

Angle Resolution	*MacroAvreageRecall*	*MacroAvreagePrecision*	*MacroF1-Score*
1.8°	0.3950	0.3927	0.3802
3.6°	0.6238	0.6387	0.6207
9°	0.9027	0.9097	0.9029
21.6°	0.9890	0.9892	0.9889

## Data Availability

Not applicable.
